# GSA MANAGING EDITORS' PERSPECTIVE ON SUBMISSION DOS AND DON'TS

**DOI:** 10.1093/geroni/igac059.438

**Published:** 2022-12-20

**Authors:** Karen Jung, Kathleen Jackson

**Affiliations:** The Gerontological Society of America, Washington, District of Columbia, United States; The Gerontological Society of America, Washington, District of Columbia, United States

## Abstract

In this presentation, the managing editors of GSA’s peer-reviewed journals will discuss how the editorial offices operate and their roles in the publishing process. The topics will include how to navigate the ScholarOne submission system, why it is important to read the Instructions to Authors, and how authors can work with the editorial offices to increase the visibility and impact of their published articles.

